# Differential Functional Connectivity in Anterior and Posterior Hippocampus Supporting the Development of Memory Formation

**DOI:** 10.3389/fnhum.2020.00204

**Published:** 2020-06-05

**Authors:** Lingfei Tang, Patrick J. Pruitt, Qijing Yu, Roya Homayouni, Ana M. Daugherty, Jessica S. Damoiseaux, Noa Ofen

**Affiliations:** ^1^Institute of Gerontology, Wayne State University, Detroit, MI, United States; ^2^Department of Psychology, Wayne State University, Detroit, MI, United States; ^3^Department of Psychiatry and Behavioral Neurosciences, Wayne State University, Detroit, MI, United States; ^4^Neurobiology Department, Weizmann Institute of Science, Rehovot, Israel

**Keywords:** memory, development, MRI, hippocampus, anterior, posterior, connectivity

## Abstract

Neuroimaging evidence suggests that the development of the hippocampus, a brain structure critical for memory function, contributes to the improvements of episodic memory between middle childhood to adulthood. However, investigations on age differences in hippocampal activation and functional connectivity and their contributions to the development of memory have yielded mixed results. Given the known structural and functional heterogeneity along the long axis of the hippocampus, we investigated age differences in the activation and functional connectivity in hippocampal subregions with a cross-sectional sample of 96 participants ages 8–25 years. We found that anterior and posterior hippocampus supported memory formation, and there was overall stability in memory-related hippocampal activation with age. Without taking account of memory outcome, direct contrast between subregions showed higher functional connectivity of anterior, compared to the posterior hippocampus, with regions in the inferior frontal and lateral temporal lobes, and higher functional connectivity of posterior, compared to the anterior hippocampus, with regions in the medial and superior frontal, inferior parietal, and occipital lobes. A direct contrast between the memory-related connectivity patterns of anterior and posterior hippocampus identified a region in the medial frontal cortex, with which anterior and posterior hippocampus was differentially functionally connected. Finally, we identified age differences in memory-related differential hippocampal functional connectivity with several frontal and visual/sensory cortices, underscoring the importance of examining age differences in the patterns of hippocampal connectivity. Moreover, the specific patterns of differential anterior and posterior functional connectivity indicate an increase in the functional specialization along the long axis of the hippocampus and a dynamic shift in hippocampal connectivity patterns that supports memory development.

## Introduction

Memory undergoes protracted development from childhood to adulthood. Two prominent brain regions, the prefrontal cortex (PFC) and the hippocampus are critical for memory formation. Previous studies have shown consistent developmental effects in the PFC supporting memory improvements from childhood to adulthood (Ghetti and Bunge, 2012; Ofen, 2012; Güler and Thomas, 2013). However, investigations on the functional development of the hippocampus portray a far less clear picture. Studies on the developmental effects of the hippocampus contributing to memory development have yielded mixed results: the function of the hippocampus and its adjacent cortices have been found to show age invariance in some cases (Ofen et al., 2007, 2012; Güler and Thomas, 2013; Shing et al., 2016), but age-related increase (e.g., Ghetti et al., 2010; DeMaster et al., 2013) or decrease (Maril et al., 2010) in others.

An important consideration when characterizing the involvement of the hippocampus is that its structure is heterogeneous, and its connectivity patterns differ drastically along the anterior-posterior axis (Poppenk et al., 2008, 2013; Strange et al., 2014). It has been shown that the granularity of encoded information increases systematically along the hippocampal long axis: anterior hippocampus preferentially encodes higher-order information, constructing memory gist, whereas posterior hippocampus preferentially encodes lower-order spatial and sensorimotor information, registering memory details (Poppenk et al., 2008, 2013; Lisman et al., 2017). In rodents, the most ventral region of the hippocampus, which is congruent to the primate anterior hippocampus, has a representational field 10 times larger than the most dorsal region, which is congruent to the primate posterior hippocampus (Kjelstrup et al., 2008). Also, studies of humans and non-human primates have demonstrated relative segregation between anterior and posterior portions of the hippocampus, such that anterior and posterior regions project to medial and lateral bands of the entorhinal cortex respectively, which are sparsely interconnected (Fanselow and Dong, 2010; Poppenk et al., 2013). Given the functional distinctions between hippocampal subregions, it is likely that the anterior and posterior hippocampus facilitate different aspects of encoding through their differential connections with the cortex.

To characterize the functional heterogeneity of the anterior and posterior hippocampus and their contributions to memory development, it is important to use methods that are sensitive and specific to the variability within this region. Functional studies on the development of the hippocampus to date, except for a recent study (Geng et al., 2019), either did not specifically segment the hippocampus from the whole brain or utilized a probabilistic atlas for segmenting the hippocampus. Moreover, when the focus is assessing anterior compared to posterior hippocampal contributions in developmental studies, the hippocampus was commonly segmented using a predefined boundary, based on* a priori* determined y-coordinates [e.g., *y* = −21 as the boundary between anterior and posterior hippocampus on the AAL hippocampal region of interest (ROI); Tzourio-Mazoyer et al., 2002]. Such a one-size-fits-all approach has been demonstrated in other applications to risk reducing the validity and sensitivity of hippocampal measures (Sandstrom et al., 2006; Wenger et al., 2014; Wisse et al., 2014). Poorly constructed ROIs may misrepresent the signals from individual hippocampi, leading to the mixing of signals of different subregions or contamination from signals of the white matter and ventricles. These misrepresentations would greatly reduce the validity and reliability of measurements of hippocampal activations (Sandstrom et al., 2006). To fully understand the developmental trajectory of hippocampal subregions, it is ideal to assess their levels of activation with ROIs specified for each individual by leveraging common anatomical expertise, as can be afforded with reliable manual segmentation of high-resolution MRI. Furthermore, most previous fMRI studies on the development of the hippocampus have adopted a standard smoothing procedure in the preprocessing steps (ranging from 6 mm to 8 mm smoothing kernels, e.g., Ghetti et al., 2010; Qin et al., 2016; Blankenship et al., 2017). While the smoothing procedure is routinely adopted to reduce the noise of the BOLD signal and generate informative clusters on the group level, it can nonetheless lead to contaminations, on an individual level, of signals from the hippocampus by surrounding cortices and ventricles, rendering the interpretations of the hippocampal ROI analyses difficult. Therefore, we opted to conduct all hippocampus-related analyses with unsmoothed fMRI data.

In addition to addressing a methodological issue for the measurement of hippocampal-specific contributions to memory development, the role of the hippocampus in the context of a larger functional network needs to be considered. Few previous studies have examined how patterns of functional connectivity with the hippocampus and surrounding medial temporal cortices differ from childhood to adulthood (Menon et al., 2005; Ofen et al., 2012; Tang et al., 2018). These few studies have consistently shown increased functional connectivity with age between medial temporal cortices and the PFC. When examined at rest, both young children (ages 4–10; Blankenship et al., 2017) and adults (Kahn et al., 2008; Poppenk and Moscovitch, 2011; Qin et al., 2016) display differential patterns of connectivity with the anterior hippocampus as compared to the posterior hippocampus. Specifically, anterior hippocampus show more functional connectivity with anterior and ventrolateral temporal cortices, while posterior hippocampus show more functional connectivity with the medial PFC, lateral parietal cortex, posterior cingulate, and retrosplenial cortices (Kahn et al., 2008; Poppenk and Moscovitch, 2011; Qin et al., 2016). In young children, hippocampal functional connectivity with the cortex shows largely overlapping developmental effects between anterior and posterior subregions, yet subtle differential developmental effects exist between hippocampal subregions and several frontal and temporal regions (Blankenship et al., 2017; Geng et al., 2019). While these studies provide insight into the development of connectivity patterns of hippocampal subregions in young children, how functional connectivity patterns of hippocampal subregions develop from childhood to adulthood remains largely unknown. Also, when examining the functional connectivity of the hippocampus in young children, previous studies have demonstrated differential developmental patterns when memory outcome was taken into consideration, compared to when it was not (task-based vs. task-free design; Geng et al., 2019). It is therefore important when characterizing the development of hippocampal functional connectivity from childhood to adulthood, to similarly compare the connectivity patterns when subsequent memory outcomes were taken into account or not.

In this study, our main goal is to assess potential age differences in the activations and functional connectivity of hippocampal subregions that specifically support memory formation. We investigated differential developmental effects in hippocampal subregions with a subsequent memory paradigm in a developmental sample. For improved validity in the signal measurement in hippocampal subregions, we individually defined hippocampal ROIs with manual segmentation of high-resolution hippocampal scans. Within these anterior and posterior hippocampus ROIs, we conducted several planned analyses to address our research question. First, we confirmed that the subsequent memory paradigm generated canonical responses from all participants in the hippocampus and across the whole brain. Then, we investigated the effect of age in the activations of hippocampal subregions. After that, we investigated differential hippocampal functional connectivity between anterior and posterior hippocampus. To address the relevance of memory outcome in the functional connectivity, we conducted two separate whole-brain connectivity analyses, first without taking into account subsequent memory and second by directly assessing subsequent memory-related connectivity patterns. Without taking into account memory outcome, we expect to identify differential connectivity of the hippocampus similar to what was identified in prior studies (Kahn et al., 2008; Poppenk and Moscovitch, 2011; Qin et al., 2016). On the other hand, when we make direct comparisons regarding the subsequent memory, we may identify differential connectivity with the medial PFC (mPFC). This prediction is based on findings of strong modulation by memory outcome of connectivity between the anterior hippocampus and the mPFC (van Kesteren et al., 2010; Preston and Eichenbaum, 2013). Finally, and critical to our main aim, we assessed age effects in subsequent memory-related differential connectivity of anterior and posterior hippocampal regions. Based on previous studies showing age-related differences in memory-related functional connectivity with posterior but not anterior hippocampus and the PFC (Menon et al., 2005; Ofen et al., 2012; Tang et al., 2018), we hypothesized there would be significant age differences in differential hippocampal functional connectivity with the PFC. Taken together, with this large sample and across a wide age range, we aimed to characterize age differences in hippocampal regional activation and connectivity patterns supporting memory performance. We predicted that assessing regional hippocampal connectivity will allow us to identify robust age differences, underscoring the importance of functional connectivity in assessing the neural basis of memory development.

## Materials and Methods

### Participants

We obtained behavior and MRI data from 96 participants ages 8–25 (16.06 ± 4.73, 53% female). Participants were recruited from the Metro Detroit area, were right-handed, had a normal or corrected-to-normal vision, not claustrophobic, and had no known history of psychiatric or neurological disorders, as reported by the participant (for adults) or their parents (for children and adolescents). Participants provided informed consent or assent as per a Wayne State University IRB-approved protocol and were compensated for their time spent in this study. Before the MRI session, the participants underwent extensive mock scanner training so that they were comfortable with the MRI environment. Data from additional 18 participants (14 children and four adolescents, 44% female) were collected but were excluded from the current analysis due to excessive head motion (average framewise displacement >0.8 mm or any single framewise displacement >6 mm).

### MRI Data Acquisition

MRI data were acquired in a 3T Siemens Verio scanner at the Harper University Hospital in Detroit, MI. T1-weighted whole-brain structural images were acquired using an MPRAGE sequence [192 sagittal slices, repetition time (TR) = 2,200 ms, echo time (TE) = 4.26 ms, flip angle = 9°, field of view = 256 mm, 192 × 256 voxels, and voxel size = 1 mm × 0.5 mm × 1 mm]. Functional images were acquired using a T2*-weighted gradient-echo sequence. Thirty sagittal slices were collected parallel to the AC-PC plane (TR = 2,000 ms, TE = 30 ms, flip angle = 90°, effective voxel size = 3.125 mm × 3.125 mm × 4.8 mm). Participants were scanned for three consecutive functional runs while performing in a subsequent memory paradigm, as described below. Each functional run consisted of 118 volume acquisitions. In addition to the T1-weighted structural and T2*-weighted functional scans, a T2-weighted high-resolution proton density-weighted turbo spin-echo (PD-TSE) sequence was included to obtain images with a high in-plane resolution for the hippocampus. Thirty coronal slices were acquired perpendicular to the long axis of the hippocampus (TR = 7,150 ms, TE = 17 ms, flip angle = 120°, the field of view = 280 mm × 512 mm, pixel bandwidth = 96 Hz/pixel, and in-plane resolution: 0.42 mm × 0.42 mm, 2 mm thick slices).

### Subsequent Memory Paradigm

**Figure 1:**
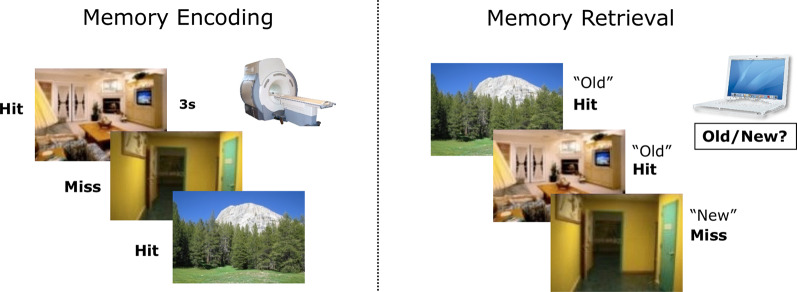
An illustration of the subsequent memory paradigm. Participants studied indoor and outdoor scenes in the scanner and completed a recognition test later outside the scanner. Based on the responses during memory retrieval, encoding trials were labeled as Hit or Miss.

All participants performed in a subsequent memory paradigm, similar to what was described in previous publications (Ofen et al., 2007; Tang et al., 2018; see Figure 1 for an illustration of the paradigm). Briefly, participants studied 120 pictures of indoor and outdoor scenes while lying comfortably in the scanner. They were instructed to respond with a button press indicating whether each picture depicts an indoor or outdoor scene. Participants were also explicitly instructed to try their best to memorize the scenes for a subsequent recognition test, in which all studied scenes will be presented along with new scenes not studied in the scanner. Approximately 15 min after the completion of the MRI session, they completed a self-paced recognition test outside the scanner (with 120 old scenes intermixed with 80 new scenes). Based on the responses during recognition, encoding trials were labeled as Hit or Miss. The stimuli set is comprised of 600 scenes, and each participant was tested with a subset of the stimuli, using three lists of 40 scenes during the study and two additional lists during recognition (foils). Different study and test lists were assigned to each participant using a pseudorandomized order. Each scene was presented for 3 s, followed by a 0.5 s fixation cross and a variable intertrial interval ranging from 0 to 12 s (negative exponential distribution). The variable intertrial interval was used to increase fMRI measurement reliability (sequence determined using optseq2^1^^,^^2^). Scenes were presented in three consecutive runs, with 40 in one run. Each run lasted for 3 min and 54 s.

For the encoding portion of the subsequent memory paradigm, we quantified the rate at which participants correctly identified the scenes as indoor or outdoor. For memory recognition, we quantified the rate at which participants correctly (Hit) or incorrectly (False Alarm, FA) recognized an image as previously studied. Recognition accuracy was calculated by adjusting Hit rates with FA rates (Hit—FA). We tested the age effect on recognition accuracy with a bivariate correlation.

### MRI Analyses

#### Processing Pipelines

**Figure 2:**
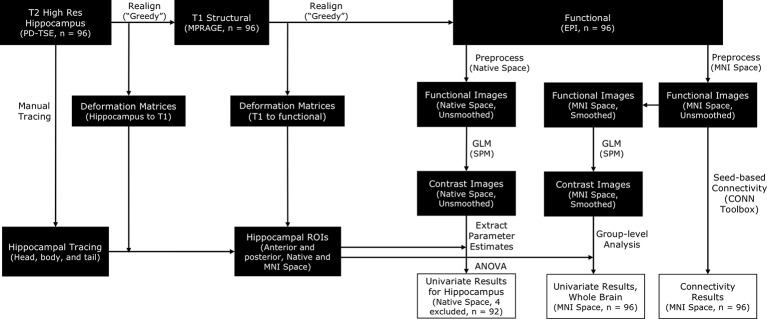
A schematic for the processing pipelines in this study. Manually traced hippocampal head, body, and tail were realigned to functional images [by applying deformation matrices derived from realigning proton density-weighted turbo spin-echo (PD-TSE) images to functional images] in order to construct individual anterior and posterior hippocampal Regions of Interest (ROIs). Functional images were preprocessed in two parallel streams, in native space or in MNI space, to facilitate different analytical needs. Analyses focusing on the hippocampus were conducted with unsmoothed images, whereas whole-brain analyses were conducted with smoothed images.

Functional MRI data were preprocessed with the SPM12 package (Wellcome Department of Imaging Neuroscience, London, UK) in MATLAB. To satisfy different analytical goals, we ran two parallel preprocessing streams. In one preprocessing stream, the functional images were preprocessed in native space for subsequent ROI analyses in the hippocampus. In the other preprocessing stream, the functional images were normalized into MNI space for subsequent whole-brain univariate analyses and connectivity analyses (see Figure 2 for a complete protocol). To preserve the specificity of signals in the hippocampus, we opted to conduct all hippocampus-focused analyses with motion-corrected but unsmoothed images.

#### Hippocampal Manual Segmentation and the Generation of Hippocampal Regions of Interest (ROI)

**Figure 3:**
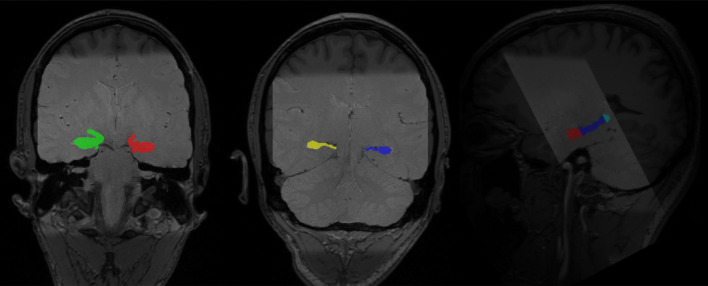
Manually segmented bilateral hippocampal head, body, and tail (in color) overlaid on top of structural images of one participant (light gray: PD-TSE high-resolution hippocampal image, dark gray: T1 MPRAGE whole-brain structural image).

To accurately capture the signals in hippocampal subregions, we followed an established protocol to segment the hippocampus into the head, body, and tail from contiguous slices obtained from the T2 high-resolution scan (Daugherty et al., 2015). Segmentation was conducted manually by four raters (AMD, RF, DM, and QY), who achieved high reliability using the protocol, before segmenting the data used for analyses in this manuscript. High reliability among raters was established on a reliability set and indicated by two-way mixed intraclass correlation coefficients [ICC(2), hippocampal head: left ≥ 0.97, right ≥ 0.97; hippocampal body: left ≥ 0.89, right ≥ 0.92; hippocampal tail: left ≥ 0.93, righ ≥ 0.92; Shrout and Fleiss, 1979], which was further confirmed by dice coefficients (Dice coefficient, hippocampal head: left ≥ 0.91, right ≥ 0.92; hippocampal body: left ≥ 0.92, right ≥ 0.91; hippocampal tail: left ≥ 0.85, right ≥ 0.83). The manual segmentation protocol was detailed in Daugherty et al. (2015) and the segmentation process was conducted in Analyze v11.0 (Biomedical Imaging Resource, Mayo Clinic College of Medicine, Rochester, MN, USA; see Figure 3 for manually segmented hippocampi overlaid on top of the structural images of one participant).

To realign hippocampal images with functional images, we adopted a standard protocol as implemented in the Automatic Segmentation of Hippocampal Subfields (ASHS) toolbox (v 1.0.0), which utilized the “greedy” algorithm^3^ to find the maximum fit between different types of images. High-resolution hippocampal images were first realigned to T1 images and then to fMRI images. The deformation matrices from these two steps were then applied to the hippocampal tracing (head, body, and tail) to move the traces to the same space as the fMRI images (see Figure 2 for a complete protocol). Anterior and posterior hippocampal ROIs were constructed separately for the left and right hippocampus per participant. The anterior hippocampal ROI was defined as the manually demarcated hippocampal head, while the posterior hippocampal ROI was defined as the manually demarcated hippocampal body and tail combined [average voxel sizes for the ROIs (3.125 mm × 3.125 mm × 4.8 mm voxels) for left anterior hippocampus: 29.68 ± 7.22, left posterior hippocampus: 32.20 ± 4.14, right anterior hippocampus: 29.74 ± 7.09, right posterior hippocampus: 31.16 ± 4.69].

#### Univariate fMRI Analyses

Preprocessed functional images were further analyzed in their respective processing space (native or MNI) with SPM12. For each of the three encoding runs, individual-level analyses included regressors of interest for subsequent memory outcomes. Regressors were modeled as subsequent hits and subsequent misses separately for high and low complexity scenes (Chai et al., 2010) to reduce possible differences related to scene complexity, which was not a focus of this investigation. Additionally, a single regressor was modeled for scenes with incorrect or no encoding (indoor/outdoor) responses to reduce possible differences due to encoding trials that were not sufficiently attended by the participant. Each encoding trial was modeled as an impulse function, convolved with a canonical model of the hemodynamic response function. Temporal derivatives were included for all conditions and were treated as regressors of no-interest. For each run, seven motion parameters were included, and outlier volumes were controlled, by including covariates calculated through the Artifact Detection Tools (ART^4^; an outlier is defined as global mean intensity >3 SD or framewise motion >1 mm).

To measure the level of neural response to the subsequent memory task and the level of neural response specifically supporting encoding success, we computed 3 contrasts of interest in each individual: (1) all Hits (vs. implicit baseline); (2) all Misses (vs. implicit baseline); and (3) Hit—Miss. Individual contrast maps and statistical maps (SPM *t*) were generated for each contrast. To confirm that the memory paradigm generated canonical subsequent memory effects, we conducted a group-level one-sample *t*-test with all individual contrast images (Hit—Miss). The resulting group-level map was thresholded at *p* < 0.001 voxel-level and corrected at *p* < 0.01 FDR cluster-level.

To understand the effects of memory outcome (Hit vs. Miss), hippocampal subregion (Anterior vs. Posterior), and hemisphere (Left vs. Right) on hippocampal activation, we extracted parameter estimates of the hippocampus for the combinations of these three factors from individual contrast images, leading to 8 (2 × 2 × 2) variables. We excluded all data from a participant if any of the eight extracted values from the participant were above 3 standard deviations from the mean of the respective variable. These exclusion criteria resulted in the exclusion of four participants and analyses for hippocampal activations were conducted with 92 participants. We conducted two ANOVAs. First, we examined memory-related activation across all participants by including the eight extracted parameter estimates as dependent variables (DVs), and memory outcome (Hit vs. Miss), subregion (Anterior vs. Posterior), and hemisphere (Left vs. Right) as independent variables (IVs), with no covariates included. After determining the overall effect of hippocampal subregions, we conducted an additional ANOVA with age as a covariate to examine age differences and approximate developmental effects in the hippocampus. To correct for multiple comparisons for the two ANOVA analyses, we set the alpha level to 0.025 (0.05/2).

#### Functional Connectivity fMRI Analyses

We next investigated the patterns of functional connectivity in the anterior and posterior hippocampus. First, we investigated differential functional connectivity between anterior and posterior hippocampus during memory encoding, regardless of memory outcomes. Then we examined differential memory-related connectivity patterns across all participants. After that, we addressed the main question of this article by investigating age effects in differential memory-related functional connectivity patterns between anterior and posterior hippocampus. Seeds of anterior and posterior hippocampal ROIs were generated based on individually demarcated tracing performed on high-resolution T2 images. Whole-brain connectivity maps with these individually defined anterior and posterior hippocampal ROIs were generated using the CONN toolbox^5^ (Susan Whitfield-Gabrieli, 2016).

To facilitate the processing of the functional data in the CONN toolbox, we normalized the functional images to the MNI space. We modeled each trial as 3 s blocks to ensure stability in the connectivity estimation and included the same conditions as in the univariate analyses, with the main focus on memory outcome (Hit vs. Miss). We extracted time-series data from the bilateral anterior and posterior hippocampus, controlling for signals in the white matter, CSF, and motion-related covariates (using the ART motion covariates as detailed above in the section describing the univariate analysis). We applied linear detrending and a high-pass filter of 0.008 Hz after regression. No de-spiking was performed. Seed-based connectivity analyses (weighted GLM, bivariate correlations) were conducted on the first level in all four hippocampal seeds.

For group-level analyses, we created several sets of models. First, we assessed differential functional connectivity between anterior and posterior hippocampal ROIs during memory encoding, irrespective of subsequent memory outcome. Second, we examined the patterns of anterior and posterior hippocampal functional connectivity that were directly related to encoding success (by assessing differential connectivity patterns in Hit compared to Miss trials, hence referred to as memory-related functional connectivity). Finally, to test possible age differences in the patterns of hippocampal subregion functional connectivity, we estimated the differential memory-related functional connectivity patterns between the anterior and posterior hippocampus, including age as a covariate of interest and controlling for covariates that were not a target of this investigation: head motion (as indexed by average framewise displacement) and recognition accuracy. The last model was the target model of our study, providing specificity in assessing age differences in differential memory-related functional connectivity between anterior and posterior hippocampus while controlling for confounds such as differences in-memory performance and motion. All models were thresholded at *p* < 0.001 on the voxel level and corrected at *p* < 0.01 FDR on the cluster level. For models that consist of double subtraction (both Anterior vs. Posterior and Hit vs. Miss), we additionally reported results with a liberal threshold (*p* < 0.005 on the voxel level and corrected at *p* < 0.05 FDR on the cluster level) as exploratory analyses.

## Results

### Behavior

**Figure 4:**
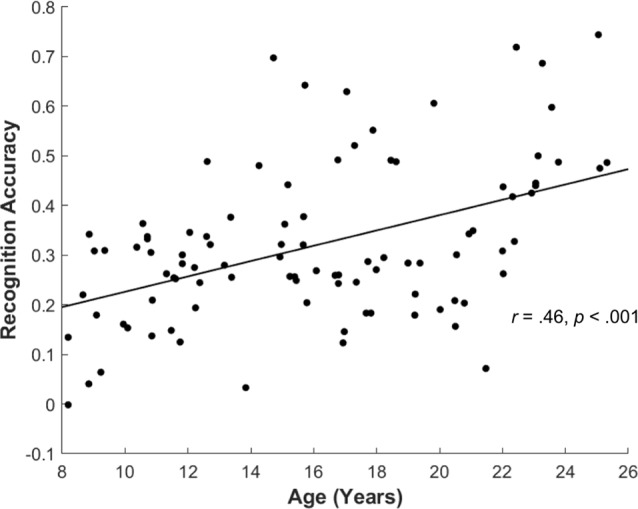
Recognition accuracy by age. Across all participants, recognition accuracy (Hit rate—False Alarm rate) showed significant increase with age (*r*_(94)_ = 0.46, *p* < 0.001).

For the subsequent memory paradigm, participants were highly accurate during the encoding task, classifying the pictures as depicting an indoor or outdoor scene (0.95 ± 0.06). The accuracy for the encoding task did not differ by age (*r*_(83)_ = −0.17, *p* = 0.11), suggesting good overall task compliance across participants. Overall, participants correctly identified 66.89 ± 15.69 items as “old” (Hit), incorrectly identified 48.39 ± 15.73 items as “new” (Miss), and incorrectly judged 20.88 ± 10.86 as “old” (FA). Participants’ recognition accuracy, defined as the difference between the Hit rate and the FA rate, was 0.32 ± 0.15 overall. Consistent with prior reports, recognition accuracy significantly increased with age (*r*_(94)_ = 0.46, *p* < 0.001; Figure 4), and both the Hit and FA rate showed correlations with age (Age × Hit: *r*_(94)_ = 0.27, *p* = 0.007, Age × FA: *r*_(94)_ = −0.27, *p* = 0.008).

### Brain Regions Relating to Encoding Success

**Figure 5:**
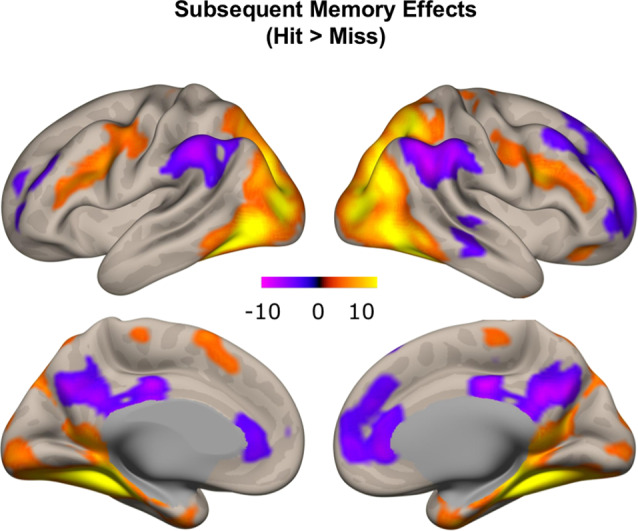
Subsequent memory effects across the whole brain. Canonical subsequent memory effects were found in a range of brain regions, including bilateral hippocampi. *p* < 0.001, FDR-corrected at *p* < 0.01.

To confirm that our study paradigm generated expected subsequent memory effects, we conducted a group-level one-sample *t*-test, combining individual subsequent memory effects (Hit—Miss) across all participants. Consistent with previous findings, we identified canonical whole-brain subsequent memory effects in our current study. We found positive subsequent memory effects in bilateral inferior frontal gyrus, parahippocampal gyrus, hippocampus, and middle occipital lobe. We found negative subsequent memory effects in middle and superior frontal gyrus (SFG), supramarginal gyrus, precuneus, and medial PFC (Figure 5, Table 1).

**Table 1:** Subsequent memory effects (Hit—Miss) across the whole brain.

Regions	BA	MNI Coordinates			*t*-value	Number of Voxels
		x	y	z		
**Positive subsequent memory effects (Hit > Miss)**
Right parahippocampal gyrus	19	34	−32	−18	15.00	35,226
Left parahippocampal gyrus	19	−32	−46	−10	13.36
Left fusiform gyrus	37	−30	−38	−18	13.10
Left hippocampus	NA	−22	−16	−20	7.16
Right hippocampus	NA	32	−12	−16	7.14
Right inferior frontal gyrus	44	44	10	28	9.38	2,499
Left cerebellum		−14	−42	−50	8.07	345
Left inferior frontal gyrus	44	−42	6	30	7.40	2484
Left precentral gyrus	6	−48	−2	56	6.71
Left inferior frontal gyrus	44	−48	18	26	6.46
Right orbital frontal gyrus	11	30	36	−14	5.78	306
Right cerebellum	NA	20	−40	−48	5.62	517
Left supplementary motor area	6	−6	12	64	4.99	393
Right paracentral gyrus	5	10	−24	62	4.68	563
Left paracentral gyrus	5	−12	−24	64	4.59
Right precentral gyrus	4	48	0	58	4.04	107
**Negative subsequent memory effects (Miss > Hit)**
Right supramarginal gyrus	40	56	−50	28	8.12	2,906
Right inferior parietal lobule	40	54	−56	42	7.68
Cingulate gyrus	24	2	−18	36	7.98	3,532
Precuneus	7	2	−64	34	7.7
Right superior frontal gyrus	10	22	56	18	7.97	6,461
	9	24	44	38	6.74
Medial prefrontal cortex	32	4	40	0	6.63
Left supramarginal gyrus	40	−56	−42	36	6.95	2,148
Left inferior parietal lobule	40	−62	−40	44	6.75
Right insula	NA	32	18	−16	6.2	202
Right middle temporal gyrus	21	54	−32	−6	5.36	719
Left middle frontal gyrus	10	−36	38	26	5.36	848
Left cerebellum	NA	−32	−80	−36	5.16	439

### Hippocampal Subregion Activations Relating to Encoding Success

Given the inconsistency in characterizing developmental effects in hippocampal activations supporting subsequent memory in previous studies, we defined hippocampal ROIs based on manual segmentation of high-resolution hippocampal images, a method that provides more robust delineation of hippocampal subregions. We first investigated whether activations in the bilateral anterior and posterior hippocampus supported encoding success and then examined whether activations in hippocampal subregions were modulated by age. We conducted two ANOVA analyses, first across all participants and then including age as a covariate. To correct for multiple comparisons for the two ANOVA analyses we conducted, we set the alpha level to 0.025 (0.05/2).

**Figure 6:**
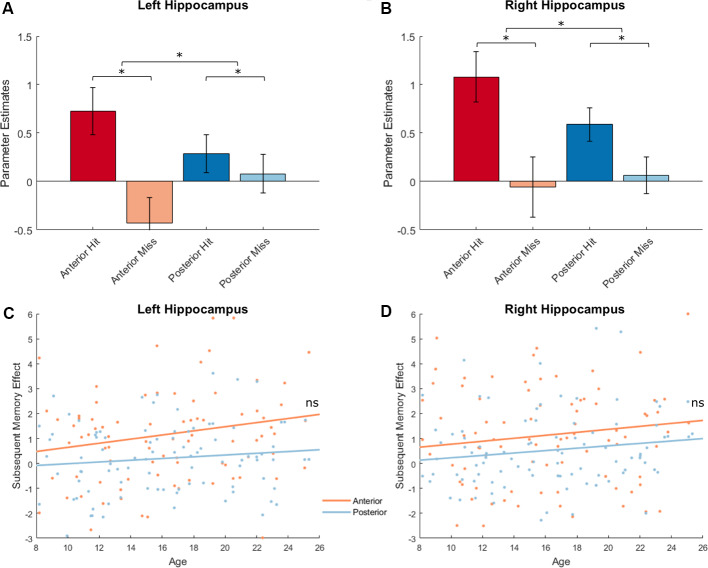
Hippocampal activations and subsequent memory effects. Top: Hippocampal activations showed a main effect of memory outcome (*F*_(1,91)_ = 19.58, *p* < 0.001), and an interaction between memory outcome and subregion [*F*_(1,91)_ = 19.86, *p* < 0.001; left hippocampus shown in **(A)**; right hippocampus shown **(B)**, *p < 0.05]. Bottom: Hippocampal activations did not show age effects. There were also no interactions between age and memory outcome, between age and subregion, or between age and hemisphere (all *p*s > 0.12; left hippocampus shown in **C**; right hippocampus shown **D**).

We extracted parameter estimates for Hit and Miss conditions from the anterior and posterior subregions separately for left and right hippocampus and conducted a memory outcome (Hit vs. Miss) × subregion (Anterior vs. Posterior) × hemisphere (Left vs. Right) ANOVA. Four participants with univariate outliers in their extracted values were excluded from analyses. We identified a main effect of memory outcome (*F*_(1,91)_ = 19.58, *p* < 0.001). Significant subsequent memory effects (Hit > Miss) were found in left anterior and right hippocampal ROIs (left anterior: 1.16 ± 2.40, *p* < 0.001, left posterior: 0.20 ± 1.39, *p* = 0.16; right anterior: 1.14 ± 2.79, *p* < 0.001, right posterior: 0.52 ± 1.50, *p* < 0.001; Figures 6A,B, plotted separately for left and right hippocampus). However, there was no subregion (Anterior vs. Posterior, *F*_(1,91)_ = 0.21, *p* = 0.65) or hemisphere (Left vs. Right, *F*_(1,91)_ = 3.04, *p* = 0.09) effect. In addition, we found an interaction between memory outcome (Hit vs. Miss) and subregion (Anterior vs. Posterior; *F*_(1,91)_ = 19.86, *p* < 0.001), such that the subsequent memory for anterior hippocampus was significantly higher than the posterior hippocampus (Anterior: 1.15 ± 2.28, Posterior: 0.36 ± 1.28, *t*_(91)_ = 4.46, *p* < 0.001). To ensure that potential differences in the signal-to-noise ratio (SNR) of hippocampal subregions do not impact on the differential subsequent memory effects identified, we extracted temporal SNR for all four hippocampal subregions based on the voxel-wise tSNR map from the ART toolbox. We found a significant difference in temporal SNR between anterior and posterior hippocampus (*F*_(1,95)_ = 713.36, *p* < 0.001). To determine whether the differential subsequent memory effects were affected by the differences in tSNR, we conducted a linear mixed model, including hemisphere (Left vs. Right) and subregion (Anterior vs. Posterior), and tSNR as fixed effects and participants as random effects. We found that subsequent memory effects were not correlated with tSNR (*t*_(364)_ = 0.22, *p* = 0.83), and the differences in subsequent memory effects by subregion remain significant after the differences in tSNR were controlled (*t*_(364)_ = 2.90, *p* = 0.004).

Next, we investigated possible age differences in hippocampal activations that support encoding success across subregions and hemispheres. Similar as before, we conducted a memory outcome (Hit vs. Miss) × subregion (anterior vs. posterior) × hemisphere (left vs. right) ANOVA and included age as a covariate. At our alpha level of 0.025, age was unrelated to hippocampal activation (*F*_(1,90)_ = 2.70, *p* = 0.10), and did not interact with effects of memory outcome (*F*_(1,90)_ = 2.43, *p* = 0.12) hemisphere (*F*_(1,90)_ = 3.10, *p* = 0.08), or subregion (*F*_(1,90)_ = 5.17, *p* = 0.03; Figures 6C,D). In the four hippocampal ROIs, no age effects were found for subsequent memory effects (age × left anterior hippocampus: *r*_(90)_ = 0.16, *p* = 0.12, age × left posterior hippocampus: *r*_(90)_ = 0.12, *p* = 0.26, age × right anterior hippocampus: *r*_(90)_ = 0.10, *p* = 0.34, age × right posterior hippocampus: *r*_(90)_ = 0.15, *p* = 0.15).

### Differential Hippocampal Functional Connectivity

To characterize the patterns of differential functional connectivity with the anterior and posterior hippocampus and how age and memory outcome modulate this pattern, we conducted several connectivity analyses using the CONN toolbox. Since there were no significant hemisphere × subregion or hemisphere × memory outcome interactions, we elected to conduct group-level connectivity analyses combining results from the left and right hippocampus to increase the statistical power to identify the patterns of functional connectivity in the anterior and posterior hippocampus.

#### Differential Hippocampal Functional Connectivity Irrespective of Memory Outcome

**Figure 7:**
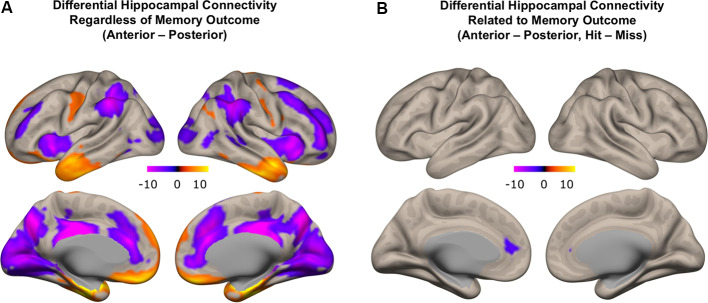
Differential functional connectivity between anterior and posterior hippocampus. **(A)** Difference in functional connectivity between anterior and posterior hippocampal subregions, regardless of memory outcome (red: higher functional connectivity with anterior compared to posterior hippocampus; blue: higher functional connectivity with posterior compared to anterior hippocampus). **(B)** Subsequent memory-related differences in functional connectivity between anterior and posterior hippocampus (blue: difference in functional connectivity with posterior compared to anterior hippocampus related to memory outcome).

We first investigated patterns of differential functional connectivity during memory encoding between the anterior and posterior hippocampus, regardless of subsequent memory outcome. We identified robust differences in the patterns of connectivity between anterior and posterior hippocampus when directly contrasting their respective connectivity maps. We observed that anterior, compared to the posterior hippocampus, showed relatively higher functional connectivity with regions in the anterior temporal lobe, orbitofrontal, inferior frontal gyrus, and premotor cortex. In contrast, posterior, compared to the anterior hippocampus, showed relatively more functional connectivity with regions in the medial and lateral frontal lobe, inferior parietal lobule, precuneus, and occipital lobes (Figure 7A; *p* < 0.01, FDR corrected; red: functional connectivity anterior > posterior hippocampus, blue: functional connectivity posterior > anterior hippocampus). These findings obtained when investigating functional connectivity patterns during encoding irrespective to subsequent memory outcome are in line with prior findings obtained when investigating differential anterior/posterior connectivity during rest in both adults (Kahn et al., 2008; Poppenk and Moscovitch, 2011; Qin et al., 2016) and children (Riggins et al., 2016). The degree to which differential anterior-posterior connectivity patterns were directly related to memory outcome, however, can only be assessed if analyses included direct measures that were gathered concerning subsequent memory outcome. Thus, we next carried out analyses to assess potentially different roles the functional connectivity of hippocampal subregions played in memory formation by employing measures of differential functional connectivity by subsequent memory outcome.

#### Differential Hippocampal Functional Connectivity Related to Memory Outcome

**Figure 8:**
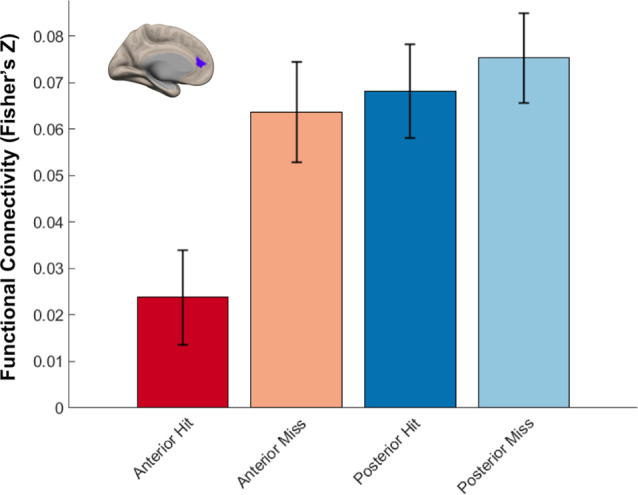
Differential hippocampal functional connectivity with medial prefrontal cortex (mPFC) related to memory outcome. Reduced functional connectivity between anterior hippocampus and mPFC was found specifically for subsequently remembered trials.

We next identified regions that showed memory-related functional connectivity with anterior compared to the posterior hippocampus. We did not find any regions that showed significant effects with our stringent threshold (*p* < 0.01 FDR corrected). Given the fact that this contrast involved double subtraction (Anterior—Posterior and Hit—Miss), we conducted additional exploratory analyses and reported the results at a more liberal threshold (*p* < 0.005 voxel-level, *p* < 0.05 FDR-corrected). Only one region was identified with this analysis, located within the mPFC. Specifically, we identified a relatively lower memory-related functional connectivity of anterior compared to the posterior hippocampus with the mPFC (Figure 7B). Follow-up analyses demonstrated low functional connectivity between anterior hippocampus and mPFC specifically for Hit trials. In contrast, high functional connectivity was observed between the anterior hippocampus and mPFC for Miss trials, and between the posterior hippocampus and mPFC for both Hit and Miss trials (Figure 8). Thus, memory-related functional connectivity was observed between the anterior hippocampus and mPFC due to lower functional connectivity between these regions for Hit trials, indicating reduced coactivation between these regions were beneficial to memory formation.

#### Differential Hippocampal Functional Connectivity With Age

**Figure 9:**
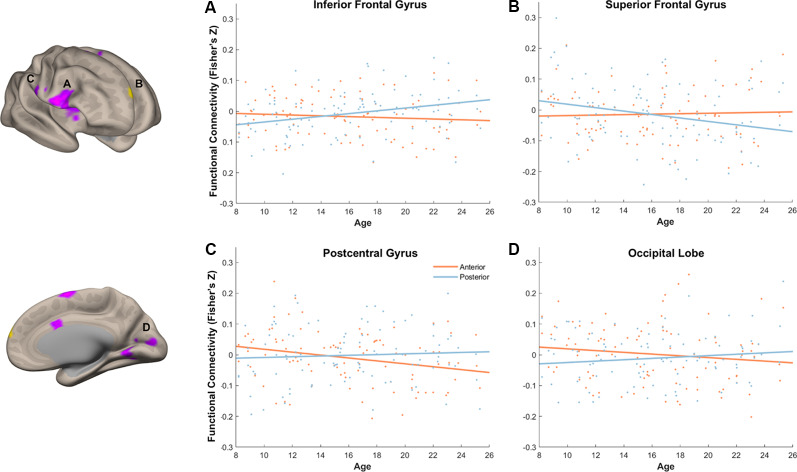
Age modulated differential memory-related functional connectivity between anterior and posterior hippocampus in the inferior frontal gyrus **(A)**, superior frontal gyrus (SFG) **(B)**, postcentral gyrus **(C)**, and occipital lobe **(D)**. With an increase of age, posterior hippocampus showed a dynamic shift in its functional connectivity pattern with the prefrontal subregions, whereas anterior hippocampus showed decreased functional connectivity to sensory and visual regions.

We next turned to examine age differences in differential memory-related functional connectivity of anterior and posterior hippocampus. To achieve this, we created additional connectivity analyses that modeled connectivity patterns per subregion and memory outcome, including age as a covariate of interest and controlling for covariates of non-interest (head motion and recognition accuracy). We identified regions that showed differential memory-related functional connectivity with anterior and posterior hippocampus that were modulated by age (Figure 9, Table 2).

**Table 2:** Age-modulated differential functional connectivity.

Regions	BA	MNI Coordinates			*t*-value	Number of Voxels
		x	y	z		
**Connectivity (Anterior—Posterior, Hit—Miss with age, *p* < 0.001, negative)**
Right inferior frontal gyrus	44	58	10	10	4.62	570
Right insula	NA	46	8	2	4.29	
**Connectivity (Anterior—Posterior, Hit—Miss with age, *p* < 0.005, negative)**
Right inferior frontal gyrus	44	58	10	10	4.62	1,473
Right putamen	NA	30	2	4	4.81
Right insula	NA	28	26	8	4.51	
Right anterior cingulate	24	2	12	24	4.4	537
Left anterior cingulate	24	−6	16	26	3.75	
Right cingulate gyrus	33	2	10	32	3.73
Right lingual gyrus	19	10	−58	−2	4.18	845
		18	−66	−2	4.12	
Left cuneus	18	−4	−90	8	3.88
Right supplementary motor area	6	6	0	68	4.58	335
		14	2	66	3.19	
Left supplementary motor area	6	−6	0	68	3.28
Right postcentral gyrus	2	50	−16	26	4.59	379
		30	−12	32	4.39	
Right supramarginal gyrus	40	44	−24	26	3.52	
**Connectivity (Anterior—Posterior, Hit—Miss with age, *p* < 0.005, positive)**
Right superior frontal gyrus	10	12	62	28	3.82	209
		10	62	20	3.45	

With our stringent threshold (*p* < 0.01 FDR corrected), we found an age-related increase in memory-related functional connectivity with IFG (Figure 9A, Table 2), such that increased functional connectivity with age was found between posterior hippocampus and IFG (*r*_(94)_ = 0.29, *p* = 0.004), but relative stability in functional connectivity with age was found between anterior hippocampus and IFG (*r*_(94)_ = −0.09, *p* = 0.39). In an exploratory analysis with a more liberal threshold (*p* < 0.005 on the voxel level, *p* < 0.05 FDR corrected), we identified additional regions that showed age-modulated differential functional connectivity, including SFG, postcentral gyrus, and occipital lobe (Figures 9B–D). Decreased functional connectivity with age was found between posterior hippocampus and SFG (*r*_(94)_ = −0.25, *p* = 0.01), but relative stability in functional connectivity with age was found between anterior hippocampus and SFG (*r*_(94)_ = 0.04, *p* = 0.69). Overall, the posterior hippocampus showed a pattern of memory-related functional connectivity with the PFC that may be reflecting a dynamic shift across development. With an increase of age, memory-related functional connectivity between the posterior hippocampus and SFG decreased, whereas memory-related functional connectivity between the posterior hippocampus with IFG increased. These effects may represent the utilization of differential memory strategies supporting encoding success across development.

Memory-related functional connectivity of the anterior and posterior hippocampus differed by age also with regions in the occipital lobe and precentral gyrus (Figures 9C,D, Table 2). Posterior hippocampus showed age invariance in its memory-related functional connectivity with regions in the postcentral gyrus (*r*_(94)_ = 0.06, *p* = 0.56) and occipital lobe (*r*_(94)_ = 0.11, *p* = 0.27). In contrast, anterior hippocampus showed an age-related decrease in functional connectivity with the postcentral gyrus (*r*_(94)_ = −0.27, *p* = 0.009), and non-significant trend in age-related decrease in functional connectivity with occipital lobe (*r*_(94)_ = −0.14, *p* = 0.18). Overall, these findings suggest that children compared to adults evince higher memory-related functional connectivity of the anterior hippocampus with visual and sensorimotor regions.

## Discussion

In this study, we examined the activation and connectivity patterns of the anterior and posterior hippocampus that supported memory formation and evaluated age differences therein. Both anterior and posterior hippocampus showed robust subsequent memory effects that were relatively stable from age 8 to 25 years. Hippocampal subregions exhibited differential functional connectivity during memory encoding irrespective of memory outcomes, such that anterior hippocampus showed stronger functional connectivity with inferior frontal gyrus and lateral temporal cortex, while posterior hippocampus showed stronger functional connectivity with the medial and superior frontal lobe, inferior parietal lobe, precuneus, and occipital lobe. We identified differential memory-related functional connectivity between anterior and posterior hippocampus with the mPFC, specifically a relative lower functional connectivity between anterior hippocampus and mPFC relating to encoding success. Differential memory-related functional connectivity of anterior and posterior hippocampus with several cortical regions was modulated by age. Overall, these age differences in connectivity patterns suggest a shift in memory-related functional connectivity between the posterior hippocampus and regions in the PFC, as well as reduced degrees of memory-related functional connectivity between the anterior hippocampus and occipital and precentral cortical regions.

Our findings of robust subsequent memory effects in the anterior and posterior hippocampus and their relative stability across age are consistent with some of the previous findings utilizing subsequent memory paradigms with either pictorial or verbal stimuli that showed age invariance in hippocampal activations (Ofen et al., 2007; Shing et al., 2016). In other studies, researchers have identified age differences in hippocampal subsequent memory activations; yet these effects were not systematically assessed concerning the anterior vs. posterior delineations of the hippocampus. Examination of these age differences in subsequent memory effects reported in previous studies suggests that they are localized in the specific regions in anterior or posterior regions of the hippocampus (Ghetti et al., 2010; DeMaster and Ghetti, 2013). Here, we attempted to investigate age differences in subsequent memory activations systematically, parsing the hippocampus to anatomically defined anterior and posterior subregions. The strength of our approach is that we extracted fMRI signals from anterior and posterior hippocampal ROIs that were generated based on the manual demarcation of these regions on specialized high-resolution hippocampal structural MR images (0.4 × 0.4 mm in-plane resolution) using a valid and reliable protocol (Daugherty et al., 2015). This approach allowed us to account for more of the individual differences in structural features in the anterior and posterior hippocampus and resulted in higher fidelity in representing fMRI signals in these regions in children and across the age range investigated here. In sum, our findings are consistent with the notion that there is relative stability in hippocampal activations supporting subsequent memory across age between middle childhood and young adulthood.

Patterns of differential functional connectivity in hippocampal subregions over a wide age range from children to adults were not reported in prior studies. We examined the patterns of functional connectivity of the anterior and posterior hippocampus when they were directly contrasted, during the encoding of scenes in preparation for a later recognition test. Anterior, compared to posterior, hippocampus showed more functional connectivity with inferior PFC and anterior temporal lobe, whereas posterior, compared to anterior, hippocampus showed more functional connectivity with the medial and SFG, inferior parietal lobule, precuneus, and occipital lobe. In a recent meta-analysis, Grady (2020) demonstrated functional differentiation along the long axis of the hippocampus, such that anterior hippocampus preferentially supports memory encoding, whereas posterior hippocampus supports memory retrieval. These results are in line with our findings of a higher overall level of activation for anterior hippocampus during memory encoding. Also, they found differential functional connectivity in the hippocampus, such that anterior hippocampus showed more functional connectivity with the ventral temporal cortex compared to the posterior hippocampus, and more functional connectivity to the posterior hippocampus with the occipitoparietal region and inferior frontal gyrus. These findings correspond to the differential functional connectivity we have identified in our current study. These differential functional connectivity effects during the task are consistent with previous studies showing differential functional connectivity with the hippocampus during rest in both children and adults (Kahn et al., 2008; Poppenk and Moscovitch, 2011; Qin et al., 2016; Blankenship et al., 2017). Together, these findings suggest that differential functional connectivity patterns along the long axis of the hippocampus may serve as an intrinsic feature that persists with age and across different task demands.

Next, we considered brain regions where anterior and posterior hippocampal connectivity differed to support encoding success. We observed differential memory-related functional connectivity of anterior compared to the posterior hippocampus with the mPFC, where decreased functional connectivity between anterior hippocampus and mPFC was indicative of successful memory encoding. This finding is consistent with prior findings of strong modulation by memory outcome of connectivity between the anterior hippocampus and the mPFC (van Kesteren et al., 2010; Preston and Eichenbaum, 2013). Thus, overall reduced connectivity of the anterior compared to the posterior hippocampus with mPFC is indicative of selective subsequent memory-related modulation of functional connectivity between these regions and a possible differentiation of hippocampal functional connectivity with one of the DMN regions. Overall, an extensive literature has highlighted the importance of the DMN in memory and other cognitive processes (Buckner et al., 2008; Christoff et al., 2009; Chai et al., 2014; Maillet and Rajah, 2016). During engaging cognitive tasks, regions in the DMN generally show reduced activation compared to rest and have been suggested to engage in the suppression of mind wandering and unrelated thoughts to facilitate task performance (Christoff et al., 2009; Maillet and Rajah, 2016). To support encoding success, it is likely that the hippocampus ramps up to promote information binding, whereas the DMN “quiets down” to suppress mind wandering and attentional shift. The mPFC, in particular, shares reciprocal structural connections with anterior hippocampus and serves as a main hub for the DMN (Buckner et al., 2008; Poppenk et al., 2013). Effective reduction in functional connectivity between anterior hippocampus and mPFC may be especially relevant for encoding success. Alternatively, the medial PFC, as suggested by previous literature, may be facilitating the utilization of pre-existing networks of knowledge, the schemas (van Kesteren et al., 2010; Preston and Eichenbaum, 2013). Interestingly, a recent study by Frank et al. (2019) shows that reduced functional connectivity between mPFC and hippocampus correlates with participants’ generalization performance, mirroring our results linking reduced functional connectivity between mPFC and anterior hippocampus to memory outcomes. Together, these findings highlight the relevance of connectivity strength between mPFC and hippocampus in facilitating regularity extraction and schema formation to support memory formation.

To address the main question of the study, which is how differential functional connectivity between anterior and posterior hippocampus supports memory development, we examined regions in the brain whose differentiation of connectivity with hippocampal subregions differed by age during successful memory encoding. We identified regions including IFG, SFG, postcentral gyrus, and calcarine sulcus that showed such effects. Specifically, memory-related functional connectivity between the posterior hippocampus and SFG decreased with age, whereas memory-related functional connectivity between the posterior hippocampus and IFG increased with age. Memory-related functional connectivity between the anterior hippocampus and both visual and sensory regions decreased with age.

Previous studies on differential hippocampal functional connectivity in young children have demonstrated a shift in the connectivity patterns between the hippocampus and PFC. For example, in children aged 4–8 years undergoing a subsequent memory task, differential functional connectivity between hippocampal subregions and IFG increased in older compared to younger children (Geng et al., 2019). In another resting-state study with children ages 4 and 6 years, anterior hippocampus showed positive functional connectivity with SFG in 6-year-olds, but negative functional connectivity for 4-year-olds (Riggins et al., 2016). The varying level of engagement between the posterior hippocampus and different PFC subregions found in the current study can be understood in the context of the PFC facilitating strategy use. During memory formation, the PFC supports spontaneous use of elaborative mnemonic strategies, and the volume of dorsolateral regions of the PFC has been shown to mediate age-related increases in strategy use in a memory task (Yu et al., 2018). It is, therefore, possible that the shifting connectivity pattern between the hippocampus and PFC subregions underlie changes in the utilization of memory strategies. Alternatively, the simultaneous increase and decrease in the functional connectivity between PFC subregions and the hippocampus can be understood in the context of correlation and anti-correlation. In our previous study investigating the development of positive and negative subsequent memory effects in the PFC (Tang et al., 2018), we have identified an age-related increase in positive functional connectivity between medial temporal lobe (MTL) and IFG, but an age-related increase in negative functional connectivity, or anti-correlation between MTL and SFG. The current findings showing an age-related increase in memory-related functional connectivity between the posterior hippocampus and IFG but age-related decrease in memory-related functional connectivity between the posterior hippocampus and SFG mirrored our previous findings, suggesting a dynamic shift in long-range functional connections between subregions of the hippocampus and subregions of the PFC.

Several related studies have examined task-related functional connectivity between hippocampal and cortical regions. For example, Lambert et al. (2017) found that children exposed to violence had greater functional connectivity between the hippocampus and ventrolateral PFC (vlPFC), which was in turn associated with worse memory performance for encoding context in the presence of a threat. Qin et al. (2014) conducted a longitudinal fMRI in 7–9-year-old children and found that the transition from the use of counting to memory-based retrieval parallels increased functional connectivity between the hippocampus and multiple prefrontal and parietal regions. Finn et al. (2010) utilized a longitudinal design and showed age effects in the functional connectivity between the hippocampus and PFC in adolescence during a working memory task, such that the PFC-hippocampal functional connectivity become specific to only high working memory load as participants mature. Together, these studies highlight the importance of the increased functional connectivity between the PFC and hippocampus in supporting the improvement of important cognitive functions.

In addition to age effects in differential functional connectivity with the posterior hippocampus, memory-related functional connectivity between the anterior hippocampus and visual and sensory regions decreased with age. To successfully encode the scene stimuli used in this study, an optimal strategy may be to extract the gist of these pictures (e.g., it is “a living room,” “a snow-capped mountain,” “a corridor with two doors”) instead of committing to memory abundant specific perceptual details contained in each scene. Consistent with the notion that the anterior hippocampus supports gist-based encoding in adults, the observed decrease in functional connectivity between the anterior hippocampus and visual/sensory regions from childhood to adulthood suggests diminished detail-oriented processing of the hippocampus in favor of high-level gist processing. Direct evidence for this interpretation can be gained in future studies with a more selective set of stimuli manipulating the predictive for successful subsequent memory based on the distinction by gist vs. detailed information.

Taken together, these findings highlight a potential developmental increase in the functional specialization along the long axis of the hippocampus. Although we utilized innovative methods to provide novel evidence for a developmental increase in differential functional connectivity in the hippocampus, we acknowledge several limitations in our study. First, while we identified an age-related difference in the functional connectivity of the hippocampus, we found only limited evidence linking such differences to the robust age differences in the memory performance. We note that age and memory performance are often colinear in the analyses of developmental data and it is, therefore, difficult to delineate separately the effect of each. In terms of the research paradigm, our study utilized indoor and outdoor scenes as stimuli. While we found no age effects in the hippocampal activation in our current study, other previous studies that used words as stimuli have found significant age effects in both anterior and posterior hippocampus (Ghetti et al., 2010; DeMaster et al., 2013), with relevance to memory performance (Sastre et al., 2016). This apparent discrepancy in the developmental effects could be due to a difference in the type of stimuli selected, with children’s development of language ability playing a role in the memory processes.

Second, in this study, we investigated the developmental effects of the hippocampus by separately examining anterior and posterior portions of the hippocampus. While functional differences have been shown between these subregions, the anterior/posterior division is a relatively crude way of delineating the hippocampus and mechanisms supporting this functional distinction remains unclear. Future studies may examine the developmental patterns of different hippocampal subfields in children to provide a clearer picture of the development of the hippocampus. In outlining the anterior and posterior hippocampus, the manual segmentation protocol we used here is highly reliable. Our goal is to assess fMRI signals and establishing high reliability in our hippocampal ROIs is important. Direct comparisons across studies are challenging, due to many methodological differences. One important aspect is the use of different segmentation protocols to define ROIs for extracting fMRI signals. Here, we emphasize that the high reliability of our manual tracing protocol is establishing an upper bound for assessing variability within the representative range of anterior and posterior hippocampus. Other approaches that include different range and sample from the most anterior and most posterior portions on the hippocampus from which our tracing protocol does not sample may represent other sources of variability that are not included in the measures reported here. Relatedly, while we have utilized innovative methods and standard resolution for developmental studies to examine hippocampal development with relatively large sample size, our ability to characterize the signals in the hippocampus is limited by the resolution of the fMRI scans. Studies with high-resolution fMRI scans may be able to provide a better understanding of the developmental effects in the hippocampus in the future. Recent continued interest has started to quantify the difference in the long-axis functional connectivity with special consideration of the subregions and encoding/retrieval dynamics (e.g., Hrybouski et al., 2019). Using ultra-high resolution structural MRI and high-resolution fMRI measures, they identified an anterior-posterior gradient in hippocampal activity when comparing encoding and retrieval. They also identified higher activity in the dentate gyrus (DG) than CA1–3 and subiculum for both memory encoding and memory retrieval. Studies like this provide additional insights into understanding hippocampal functions of specific subfields and subregions. While conducting fMRI research of higher resolution on children may prove to be challenging, future studies may explore ways to balance high resolution with good imaging quality, to provide a better understanding of the developmental effects in the hippocampus.

Third, it is important to note that the findings we present are from analyses conducted using a cross-sectional sample. Although cross-sectional samples have been widely used to provide information on memory development, the developmental differences identified with cross-sectional studies should be interpreted with caution. Only with longitudinal data would it be possible to show the relations between individual neural and behavioral changes over time. Future research using the longitudinal design is needed to validate findings from cross-sectional investigations such as this.

Fourth, in conducting the connectivity analyses, we elected for a seed-based connectivity approach, where correlations of HRF-weighted time courses were calculated in reference to hippocampal ROIs. While we have a relatively large sample size, our run length of 4 min and the rapid event-related design may limit statistical power the to detect differences between encoding conditions. Also, with this method of choice, we assumed comparable hemodynamic response across various cortical and subcortical regions, which is further complicated by contrasting different conditions. Future studies may utilize methods that do not rely on the assumption of canonical HRF to examine these connectivity effects (e.g., Hrybouski et al., 2019). Also, as is widely known in developmental fMRI research, head motion can significantly impact fMRI signals, which in developmental studies could be misinterpreted as age effects (Power et al., 2012). We took multiple measures to mitigate the effect of motion, including mock scanner training, data screening, and data scrubbing. For data scrubbing, we utilized stringent criteria with ART in both univariate and connectivity-based analyses and have additionally included motion as a covariate in the group-level connectivity analyses. While we have taken these measures to reduce the effect of motion, we acknowledge that the residual effects could still confound some of the age effects in functional activation and connectivity as we have identified.

Finally, although we utilized a relatively large sample size to conduct this study, we acknowledge that for fMRI research in general, the reliability of individual measurements remains modest and the reliability of a commonly used event-related paradigm, such as the subsequent memory paradigm is unknown. Future studies should aim to quantify the level of reliability in a developmental sample to facilitate the understanding of true developmental differences.

In sum, we systematically investigated the development of activation and connectivity patterns of the hippocampus from middle childhood to adulthood. We found that while the level of activation in the hippocampus remained relative stable with age, anterior and posterior hippocampus showed distinct connectivity patterns supporting encoding success, which exhibited robust modulation by age. The age-related increase in differential functional connectivity with the hippocampus suggests an increased specialization of the hippocampus along its long axis and a shift in positive and negative functional connections with the neocortex to support effective memory encoding.

## Data Availability Statement

The datasets generated for this study are available on request to the corresponding author.

## Ethics Statement

The studies involving human participants were reviewed and approved by Wayne State University. Written informed consent to participate in this study was provided by the participants’ legal guardian/next of kin.

## Author Contributions

NO and LT designed the study. LT, QY, RH, and NO collected the data. LT and PP conducted fMRI analyses. QY, AD, and RH performed structural MRI analyses. NO supervised all aspects of the study. LT and NO drafted and LT, JD, AD, and NO revised the manuscript.

## Conflict of Interest

The authors declare that the research was conducted in the absence of any commercial or financial relationships that could be construed as a potential conflict of interest.

## Footnotes

^1^http://surfer.nmr.mgh.harvard.edu/

^2^https://surfer.nmr.mgh.harvard.edu/optseq/

^3^https://sites.google.com/view/greedyreg/documentation

^4^http://www.nitrc.org/projects/artifact_detect/

^5^http://www.nitrc.org/projects/conn
